# Dover’s Powder Modified

**Published:** 1840-10

**Authors:** 


### DOVER'S POWDER MODIFIED.

Dr. Townsend, of Wheeling, has sent to Dr. Drake a note, in which he recommends two new ingredients in the formula for this popular compound—nitrate of potash and the powdered root of the sanguinaria Canadensis. Many of our brethren in the West, have long been accustomed to substitute the former of these articles for the sulphate of potash, but have not dispensed with the Ipecacuanha. Here is the Doctor's recipe: Opium and the powdered root of the sanguinaria, each one grain, nitrate of potash eight grains, mixed intimately by trituration. This preparation he has used, instead of the officinal, since the year 1816. The following are his pharmaceutic and practical remarks concerning it:

"It is said in our Dispensatories that the only use of the sulphate of potash is, to reduce the opium to an impalpable powder by the hardness of its crystals. I dry the opium and then pulverize it until it all passes through a fine seive. This enables me to use the nitrate of potash, which is a diuretic salt of long established value, and always admissible when the Dover's powder is proper.

Instead of the ipecacuanha of the old preparation, I have substituted the pulverized root of the *sanguinaria canadensis*, which is as good a diaphoretic as the the ipecac., at the same time that it is an alterative of nearly as high powers as mercury. My Dover's powder, then, does not contain any thing in the old preparation but the opium.

"I am aware that many are opposed to every thing new. But let such look at the long list of diseases enumerated by Professor Tully, in which sanguinaria has been found highly beneficial as a deobstruent or alterative; and they may be enabled to appreciate its value as a substitute for ipecacuanha in the composition of Dover's powder; and instead of a useless innovation, they may find it to be a real improvement. There is now no question that, as a diaphoretic, for which Dover's powder is so frequently employed, the sanguinaria will do all that ipecacuanha is capable of doing. If, then, sanguinaria possesses other and valuable properties, not possessed by the ipecacuanha but calculated to fill the indications for which Dover's powder is usually prescribed, it must be obvious that it will render the new preparation a better article, and consequently deserving of the attention of the medical profession.

"I might, from the facts already known of the *modus operandi* of the sanguinaria, go on to show, theoretically, that this modification of the Dover's powder is calculated to make it a very superior article to the old preparation. But I am contented with using it in my own practice, and with giving this simple notice, that those of the profession who think proper may avail themselves of any advantage this preparation can afford. The sanguinaria is the growth and product of our own country. This alone is by no means an unimportant consideration, inasmuch as it will assist, as far as it goes, in rendering us independent of other and foreign countries for a necessary article." D.

